# Perceived integration as a gratification facilitator: psychological drivers of students’ continuance intention in blended learning

**DOI:** 10.3389/fpsyg.2026.1857351

**Published:** 2026-08-31

**Authors:** Danyang Li, Meiling Liu, Minyu Shen

**Affiliations:** 1 Office of Development and Planning, Southwestern University of Finance and Economics, Chengdu, China; 2 School of Business Administration, Southwestern University of Finance and Economics, Chengdu, China; 3 School of Management Science and Engineering, Southwestern University of Finance and Economics, Chengdu, China

**Keywords:** blended learning, continuance intention, gratification, perceived integration, perceived overload, uses and gratifications theory

## Abstract

What sustains students’ intention to continue using educational technology? In blended learning, prior research identifies satisfaction as a key predictor of continuance intention but often treats it as a single construct and pays limited attention to the environmental conditions that enable different forms of gratification. This study extends Uses and Gratifications Theory (UGT) in two ways. First, it introduces perceived integration as an environmental antecedent of gratification. Perceived integration captures the coherence students perceive between online and offline learning components and is theorized as a gratification facilitator. Second, the study distinguishes content satisfaction, process satisfaction, and social satisfaction as separate forms of gratification and examines these gratification processes together with perceived overload informed by Cognitive Load Theory. We analyzed survey data from 327 Chinese university students using partial least squares structural equation modeling. Perceived integration strongly predicted all three forms of satisfaction, with *β* values ranging from 0.780 to 0.817. Content and process satisfaction significantly predicted continuance intention, with *β* values of 0.348 and 0.339, respectively, whereas social satisfaction did not. System functionality overload, information overload, and social overload did not significantly predict continuance intention. Together, these results show that gratification structures vary across technology contexts and differ from patterns reported in social media research. They also identify integration quality, rather than feature richness, as a key design priority for blended learning.

## 1 Introduction

Understanding what motivates students to continue to use technology-mediated learning environments is a fundamental question in both educational psychology and information systems research (Ryan and Deci, 2000; Bhattacherjee, 2001). In educational contexts, this question becomes particularly salient as institutions increasingly adopt blended learning approaches that combine face-to-face instruction with online components (Garrison and Kanuka, 2004). While initial adoption of educational technologies has been extensively studied through frameworks such as the Technology Acceptance Model (TAM) (Davis, 1989) and the Unified Theory of Acceptance and Use of Technology (UTAUT) (Venkatesh et al., 2003), the psychological factors associated with students’ continuance intention remain less clear. This distinction matters because initial acceptance and continuance intention may have different antecedents (Bhattacherjee, 2001). The Uses and Gratifications Theory (UGT), originally developed in mass communication research (Katz et al., 1974), offers a promising framework for understanding continuance intention with educational technology. UGT posits that individuals actively select media to fulfill specific psychological needs, and that the gratifications obtained from media use determine continuance intention (Rubin, 1985). This active audience perspective aligns well with contemporary understanding of learners as self-regulated agents who make deliberate choices about their learning strategies and environments (Zimmerman, 2002).

However, applying UGT to educational technology contexts raises important theoretical questions. In entertainment and social media research, social gratifications (e.g., relationship maintenance and social interaction) consistently emerge as strong predictors of continued use (Ku et al., 2013; Gan and Wang, 2015). Yet educational contexts differ fundamentally from entertainment contexts in their goal structures, motivational orientations, and expected outcomes. Whether the gratification patterns observed in social media research generalize to educational technology settings remains an open empirical question with significant theoretical implications for both UGT and educational psychology.

A second theoretical consideration concerns cognitive load. Cognitive Load Theory (CLT) suggests that excessive information processing demands can impair learning and potentially lead to disengagement (Sweller, 1988; Kirschner, 2002). In technology-rich learning environments, students may experience multiple forms of overload: system functionality overload from complex platform features (Karr-Wisniewski and Lu, 2010), information overload from abundant learning resources (Misra and Stokols, 2012), and social overload from excessive interaction demands (Maier et al., 2015). How these overload experiences relate to continuance intention represents an important but understudied question at the intersection of cognitive and motivational psychology. UGT and CLT thus play complementary roles in our framework: UGT captures the benefit side of the blended learning experience by explaining the gratifications that pull students toward continued use. By contrast, CLT captures the cost side by explaining the overload experiences that may push them away. Examining both within a single model provides a more complete account of continuance intention than either theory alone.

Blended learning, defined as the strategic integration of face-to-face instruction with online learning components, has become a cornerstone of contemporary higher education, a trend accelerated by the global pandemic (Garrison and Kanuka, 2004; Kang et al., 2021). By combining the immediacy of classroom interaction with the flexibility of digital resources, blended learning offers students personalized learning paths, enhanced accessibility, and opportunities for self-directed skill development (Dziuban et al., 2018; Yang and Ogata, 2023). These characteristics make blended learning an ideal context for examining how environmental integration shapes gratification and continuance intention. The blended learning landscape is itself evolving rapidly. Generative AI tools such as ChatGPT are increasingly being incorporated into blended learning environments in higher education (Lee et al., 2024; Liang et al., 2026). As the number and heterogeneity of components within blended environments grow, it becomes more, not less, pressing to examine how coherently these components fit together and how that coherence shapes students’ willingness to continue.

Research on blended learning has evolved along two identifiable streams. The first stream examines initial adoption and acceptance, applying and extending frameworks such as TAM and UTAUT (Padilla-Meléndez et al., 2013; Khechine et al., 2014; Al-Azawei et al., 2017; Li et al., 2022; Yu et al., 2023). Despite differences in samples and model extensions, these studies converge on a common conclusion: beliefs about a technology’s usefulness and ease of use, together with individual differences such as gender, age, learning style, and self-efficacy, shape students’ willingness to adopt blended learning. What also unites this stream, however, is its focus on the pre-adoption or early-adoption stage. It explains why students begin using blended learning, but not why they continue. The same focus on initial acceptance appears in research on other pedagogical innovations, such as Chinese university students’ intention to accept inquiry-based teaching (Hu et al., 2024).

The second stream shifts attention to post-adoption experiences, centering on student satisfaction as the focal outcome. Some of these studies examine satisfaction and its drivers as ends in themselves, highlighting teacher-student interaction, curriculum design, and perceived usefulness (Abbas, 2018; Deng et al., 2022). A smaller set takes the further step of linking satisfaction to continuance intention, whether in blended settings (Lin et al., 2016; Yang et al., 2023) or in fully online ones (Ye et al., 2022; Ye et al., 2023). Although this stream advances research beyond initial adoption, it still provides a limited account of why students continue to use blended learning. In particular, satisfaction is often treated as a single overall construct, its environmental antecedents remain underexamined, and potential negative experiences associated with blended learning have received limited attention.

Taken together, prior research leaves four specific gaps that motivate the present study. First, at the construct level, although integration is the defining characteristic of blended learning, perceived integration has rarely been conceptualized, measured, or tested as a determinant of the post-adoption experience. It refers to students’ perceptions of how coherently online and offline components work together. Second, theoretically, UGT research has concentrated on the consequences of gratifications while paying little attention to their environmental antecedents, including the design characteristics of the media environment that enable gratifications to arise. Third, the contextual transferability of existing findings remains uncertain. Social media research has often identified social gratifications as strong predictors of continued use, but this pattern cannot be assumed to generalize to educational settings, whose goal structures and motivational orientations differ fundamentally. Finally, empirical research on students’ continuance intention has largely overlooked potentially negative experiences in blended learning, including system functionality overload, information overload, and social overload.

The present study addresses these gaps by examining the relationships between perceived integration and three satisfaction dimensions (content satisfaction, process satisfaction, and social satisfaction), and between these dimensions and continuance intention. We also investigate the relationships between perceived overloads and continuance intention. Our study makes three primary contributions to the relevant literature:

First, we introduce and validate perceived integration as a critical antecedent of gratification in technology-mediated learning. While traditional UGT research focuses primarily on gratifications sought and obtained, our findings demonstrate that perceived integration functions as an effective gratification facilitator, strongly predicting content, process, and social satisfaction alike (β ranging from 0.78 to 0.82). This extends UGT by highlighting the role of environmental design characteristics in shaping students’ gratification experiences.

Second, we reveal a distinct prioritization of gratifications in educational technology contexts. Content satisfaction and process satisfaction (reflecting cognitive and instrumental gratifications) significantly predict continuance intention, while social satisfaction shows no significant effect. This pattern challenges assumptions derived from social media research and suggests that gratification structures are context dependent.

Third, we contribute to the integration of UGT with CLT by examining whether perceived overloads impede continuance intention. Our results suggest that contemporary learners may have adapted to information-rich environments, raising questions about boundary conditions of cognitive load effects.

The remainder of this paper is structured as follows. Section 2 presents the conceptual framework and research hypotheses based on the UGT and CLT. Section 3 describes the methodology and reports the empirical results. Section 4 discusses the findings. Section 5 discusses the theoretical and practical implications of our results. Section 6 acknowledges study limitations and suggests directions for future research. Finally, Section 7 concludes by summarizing key findings.

## 2 Conceptual framework and research hypotheses

This section outlines the conceptual framework of our study, which investigates the factors influencing students’ continuance intention of blended learning. Following the distinction drawn by Imenda (2014), we position our framework as a conceptual rather than a theoretical framework. We integrate constructs from UGT and CLT into a testable model for the blended learning context. UGT informs the gratification-related constructs, while CLT informs the overload-related constructs. Our theoretical contribution accordingly lies in extending UGT with an environmental antecedent (perceived integration) and in examining gratification and overload factors within a common conceptual framework, not in proposing a new theory.

**Figure 1:**
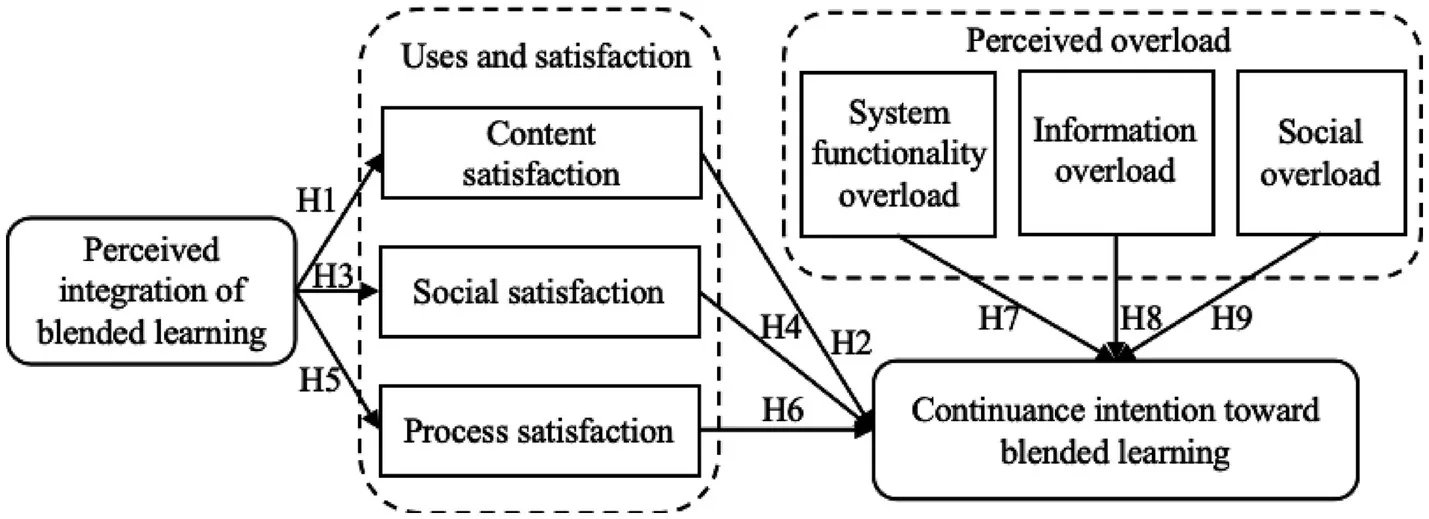
Proposed research framework (H1–H9 refer to the 9 hypotheses).

We begin by introducing UGT as a framework for understanding the gratification-related variables in Section 2.1, followed by a discussion of the key constructs and variables in Section 2.2. Finally, we present our research hypotheses, which form the basis of our empirical investigation in Section 2.3. The overall conceptual framework containing these hypotheses is depicted in Figure 1.

### 2.1 Uses and gratifications theory (UGT)

The UGT, originally introduced by Katz et al. (1974), provides a valuable framework for understanding user engagement with media. UGT posits that individuals actively select and use media to fulfill specific needs, emphasizing the audience’s agency in media consumption (Rubin, 1985). This theory has been instrumental in elucidating the motivations behind media engagement and has been extensively applied to interpret user behaviors in social media contexts, including adoption, continuous usage, and utilization of specific features (Lee and Ma, 2012; Ku et al., 2013; Guo et al., 2016). More recently, UGT has been extended to emerging intelligent technologies. Lin and Ng (2025) used UGT to examine the gratifications and concerns associated with generative AI, while Yu et al. (2024) incorporated UGT into an analysis of ChatGPT satisfaction and continued use in higher education. These studies demonstrate the continuing relevance of UGT for analyzing engagement with evolving technology-mediated learning environments.

While UGT has been widely employed in social media research, its application to blended learning contexts offers a novel perspective. The parallels between social media and blended learning platforms, such as interactivity, content sharing, and user-generated content, make UGT a suitable framework for investigating the satisfaction components that may influence students’ continuance intentions. Therefore, we use UGT to distinguish three forms of satisfaction and examine their relationships with perceived integration and continuance intention in blended learning environments.

Grounded in UGT (Katz et al., 1974), we distinguish three satisfaction dimensions reflecting different forms of gratification: content satisfaction, process satisfaction, and social satisfaction. We examine the relationships between perceived integration and each satisfaction dimension, and between each satisfaction dimension and continuance intention.

While UGT has proven valuable for understanding media selection and use, the theory has traditionally focused on the relationship between gratifications sought, gratifications obtained, and continued use. Less attention has been paid to the antecedents of gratification, that is, what environmental or design characteristics enable users to experience gratification in the first place. In blended learning contexts, we propose that the degree of integration between online and offline components represents such an antecedent. When learners perceive a seamless, coherent integration between different learning modalities, they may be better positioned to fulfill their needs for content acquisition, process engagement, and social interaction. We thus conceptualize perceived integration as a *gratification facilitator*: an environmental characteristic that enables or enhances the gratification experience. This conceptualization extends UGT by incorporating the role of media environment design in shaping gratification processes.

### 2.2 Key constructs and variables

#### 2.2.1 Dependent variable: continuance intention toward blended learning

Continuance intention refers to students’ willingness to continue using blended learning in the future and to recommend it to others. Because all participants had completed at least one blended course, this intention concerns continued use after prior experience. In the present context, instructors typically arrange the use of blended learning components during a course, so such use is largely mandatory. The dependent variable therefore captures students’ stated intention regarding future use rather than their compliance with the requirements of a particular course.

#### 2.2.2 Independent variables - perceived integration

Perceived integration in blended learning refers to the degree to which students perceive the seamless combination of online and offline learning activities. It encapsulates the learner’s perception of the cohesiveness and effectiveness of the educational process across different platforms, binding digital and traditional learning experiences. When learners perceive a high degree of integration, they are expected to experience increased satisfaction and a more holistic educational journey that caters to their diverse learning needs.

The significance of perceived integration lies in its potential to transform the educational landscape by fostering an environment where technology and traditional teaching methods coalesce to create a rich, interactive, and continuous learning experience. This integrated approach can lead to higher levels of learner engagement, satisfaction, and ultimately, a greater propensity for continued use of blended learning platforms.

Perceived integration facilitates gratification by creating environmental conditions that support users’ need fulfillment through media engagement. From a UGT perspective, perceived integration can be understood as an environmental characteristic that facilitates gratification by enabling students to more effectively fulfill their psychological needs through the learning platform. When online and offline learning components are well-integrated, students can seamlessly access content, engage with learning processes, and participate in social interactions across modalities. This theoretical positioning distinguishes perceived integration from the gratifications themselves; rather than representing a type of satisfaction, integration represents a structural property of the learning environment that shapes the conditions under which gratifications can be experienced.

#### 2.2.3 Independent variables – perceived overload

Perceived overload in blended learning denotes a cognitive state where the influx of information and system functionalities surpasses students’ capacity to process effectively (Karr-Wisniewski and Lu, 2010). This phenomenon manifests in three primary forms:

1. System functionality overload (SFO): Occurs when the features of learning platforms are ill-suited to tasks or perceived as overly complex.
2. Information overload (IO): Sets in when the volume of information to be processed exceeds an individual’s capacity.
3. Social overload (SO): Characterized by disruption caused by excessive social demands, consuming attention and energy.

In the blended learning context, students may encounter these challenges through online platforms like Massive Open Online Courses (MOOCs) and the ChaoXing learning system. Both online platforms and traditional classrooms can resemble virtual societies where abundant social interactions can lead to a crowded online space, imposing significant cognitive and temporal burdens on students.

Understanding and addressing these dimensions of perceived overload is essential for developing effective blended learning strategies that mitigate negative impacts and enhance the overall educational experience.

#### 2.2.4 Satisfaction dimensions

Our model includes three satisfaction dimensions informed by UGT (Katz et al., 1974). These dimensions reflect different forms of gratification and are examined in relation to perceived integration and continuance intention:

- Content satisfaction (CS): Refers to the fulfillment derived from the quality and relevance of the learning materials provided in the blended learning environment.
- Social satisfaction (SS): Pertains to the gratification obtained from social interactions and community building within the blended learning context.
- Process satisfaction (PS): Relates to the enjoyment and perceived benefits of engaging with the blended learning process itself.

### 2.3 Research hypotheses

#### 2.3.1 Relationships among perceived integration, satisfaction, and continuance intention

As previously discussed, we examine the relationships between perceived integration and three satisfaction dimensions informed by UGT, and between these dimensions and continuance intention. The rationale for each hypothesized relationship is presented below:

*Content satisfaction* serves as a primary driver for the adoption of blended learning. Research has demonstrated the substantial potential of blended learning in enhancing student engagement across both physical and digital learning environments (Venkatesh et al., 2003; Wu and Liu, 2013). By seamlessly integrating offline and online modalities, blended learning elevates student participation through strategies such as interactive multimedia resources and real-time feedback, fostering a more immersive and adaptive learning experience. As students perceive higher utility in this integrated approach, they exhibit increased willingness to invest time, display greater patience, and maintain enthusiasm, thereby mitigating boredom and cultivating a receptive mindset towards learning materials. The stronger the alignment and cohesion between online and offline components in blended learning, the greater the sense of content satisfaction experienced by students. Thus, we propose the following hypotheses:

*H1*: Perceived integration of blended learning positively impacts students’ content satisfaction.

*H2*: Content satisfaction positively impacts students’ continuance intention toward blended learning.

*Social satisfaction* in the context of blended learning reflects the degree of fulfillment derived from interactions among students, as well as between students and educators, facilitated by the learning platform. Enabled by mobile internet technologies, these interactions transcend traditional spatial and temporal boundaries and foster an environment where learners can seamlessly engage with platforms and peers at their convenience. This enhanced accessibility promotes efficient communication and consultation and nurtures a supportive learning community that effectively addresses students’ social interaction needs. Consequently, heightened perceptions of online-offline integration correlate with increased social satisfaction within the blended learning milieu. We therefore hypothesize:

*H3*: Perceived integration of blended learning positively influences students’ social satisfaction.

*H4*: Social satisfaction positively impacts students’ continuance intention toward blended learning.

*Process satisfaction*, also known as hedonic satisfaction, focuses on the enjoyment experienced during the use of blended learning, encompassing aspects such as engagement, interaction, and skill enhancement. Students perceive the integration of online and offline components in blended learning, where social integration allows for real-time communication with like-minded peers, contributing to a pleasurable and participatory learning experience. The convenience afforded by blended learning’s integration leads to a positive learning experience, often motivating students to prioritize blended learning in their schedules. During leisure time, they may engage with it for online learning, social interaction, browsing newly released educational resources, or simply as a means of relaxation, thereby achieving hedonic satisfaction. The stronger the perception of integration, the greater the sense of process satisfaction in using blended learning. Thus, we posit:

*H5*: Perceived integration of blended learning positively affects students’ process satisfaction.

*H6*: Process satisfaction positively impacts students’ continuance intention toward blended learning.

#### 2.3.2 Impacts of perceived overload on students’ continuance intention toward blended learning

Perceived overload is recognized as a significant factor that can negatively influence user behavior in the context of information technology usage, potentially leading to actions such as lurking, blocking, ignoring, and abandonment (Moore, 2000; Maier et al., 2015; Lee et al., 2016). This construct is critical in analyzing the factors affecting the continuance intention toward blended learning environments, as it can result in detrimental outcomes such as disengagement and discontinuation.

We explore the negative impact of perceived overload on students’ willingness to use blended learning by examining three dimensions: system functionality overload, information overload, and social overload. The rationale for using these three variables is described as follows.

*System functionality overload* reflects the degree of students’ perception to which the functionalities provided by blended learning platforms exceed their needs. The functionalities and system features of these platforms can indeed attract students and elevate their engagement with digital learning environments (Lin et al., 2016). However, an overabundance of features can potentially induce student fatigue and dissatisfaction. For instance, the integration of overly complex social media functionalities can lead to negative outcomes associated with information technology usage (Oviatt, 2006). Moreover, according to the CLT, superfluous features can divert students’ attention and impose a perceptual burden (Kirschner, 2002). The difficulty in mastering all the functionalities of blended learning platforms can result in inconvenience and student attrition. When the learning curve or complexity of use outweighs the benefits offered by the platform, students may perceive system functionality overload, leading to impatience and a negative attitude, potentially causing them to discontinue use. Therefore, we hypothesize:

*H7*: System functionality overload will have a negative impact on students’ continuance intention toward blended learning.

*Information overload* occurs when the volume of information received through blended learning platforms far exceeds one’s capacity to process it. As Misra and Stokols (2012) note, typical signs of information overload include mental and emotional fatigue, stress, anxiety, and a sense of helplessness. When the influx of learning tasks and information from the platform becomes overwhelming, students may feel inundated and unable to cope, leading to a perception of information overload. This can challenge their limited information processing capabilities, causing feelings of losing control, confusion, and decision regret, potentially leading to aversion and discontinuation of blended learning use. Hence, we propose:

*H8*: Information overload will have a negative impact on students’ continuance intention toward blended learning.

*Social overload* happens when one feels that maintaining online social interactions in blended learning requires excessive time and effort. As Maier et al. (2015) point out, social overload is a factor leading to social fatigue, defined as the exhaustion associated with blended learning-related activities. When students encounter an overwhelming number of virtual demands from online interactions, such as frequent responses to peers’ posts or participation in discussions, they may exhibit more negative emotions. Excessive or unnecessary social interactions can distract students from their regular work and study, causing mental fatigue and reluctance to continue using the platform. Therefore, we hypothesize:

*H9*: Social overload will have a negative impact on students’ continuance intention toward blended learning.

## 3 Research methodology

### 3.1 Participants and procedure

This study engaged participants from several national key universities in Chengdu, Sichuan, China, who were familiar with blended learning and had completed at least one blended course. In the participating universities, blended learning is course embedded. During a blended course, instructors arrange the use of online components, such as MOOCs and the Chaoxing learning system, so this use is largely mandatory for enrolled students. The focal outcome of this study, continuance intention, measures students’ stated willingness to continue using blended learning in the future and to recommend it to others, as reflected in items CI1–CI3 in Table 1, rather than their compliance with current course requirements. Section 4.4 discusses the implications of this quasi-mandatory usage context for interpreting the findings.

**Table 1:** Latent factors and survey items.

Constructs	Items	Statement	Sources
Continuance intention (CI)	CI1	I will continue to use blended learning.	Venkatesh et al. (2003)
	CI2	I intend to continue using blended learning in the future.	
	CI3	I would recommend blended learning to others.	
Perceived integration (PI)	PI1	The integration of online resources with offline classroom content facilitates comprehensive knowledge acquisition and understanding.	Yu et al. (2023)
	PI2	Online learning activities complement offline classroom discussions and practical sessions, enhancing my learning experience.	
	PI3	Blended learning expands my learning space and time beyond the confines of the classroom.	
Content satisfaction (CS)	CS1	Blended learning platform offers a diverse range of learning materials.	Katz et al. (1974)
	CS2	Blended learning meets my individual learning needs.	
	CS3	High-quality resources on the blended learning platform significantly promote a thorough understanding of the course content.	
	CS4	The platform’s additional resources are beneficial for my studies and reference.	
Social satisfaction (SS)	SS1	Blended learning is conducive to self-improvement.	Katz et al. (1974)
	SS2	Online activities such as discussions and group assignments help me build a learning community.	
	SS3	Blended learning enhances collaborative abilities among students.	
Process satisfaction (PS)	PS1	I am willing to engage in various interactions within the blended learning environment, including those between students, teachers, and the platform.	Katz et al. (1974)
	PS2	Using blended learning is an enjoyable experience that often leaves me feeling excited and comfortable.	
	PS3	Blended learning improves my learning skills and efficiency, particularly in acquiring and utilizing information.	
	PS4	Blended learning increases my level of engagement and enthusiasm for learning.	
System functionality overload (SFO)	SFO1	The complexity of the blended learning platform and software hinders my learning progress.	Karr-Wisniewski and Lu (2010), Yu et al. (2023)
	SFO2	Mastering all features of the blended learning platform is challenging, causing inconvenience in usage.	
	SFO3	I feel the need to reduce time spent on the blended learning platform to avoid feeling overwhelmed.	
Information overload (IO)	IO1	It is difficult for me to filter key learning content from the vast amount of information on the platform.	Misra and Stokols (2012)
	IO2	The volume of learning tasks and information on the platform distracts me from focused learning.	
	IO3	Feeling overwhelmed by the need to learn independently on the platform.	
	IO4	Only a small portion of the platform’s resources align with my interests.	
Social overload (SO)	SO1	Social interactions such as discussions and peer reviews on the platform consume a significant amount of my learning time.	Maier et al. (2015)
	SO2	To enhance learning efficiency, I feel the need to reduce my participation in social activities on the blended learning platform.	
	SO3	Focusing on peers’ learning statuses or issues takes up too much energy, affecting my own learning tasks.	

To recruit participants, we reached out to educators responsible for blended learning courses and requested their assistance in distributing our online survey to their students. Participation was voluntary, and no compensation was offered to respondents. The survey was conducted over a 10-day period from June 18 to June 27, 2024. The survey yielded 417 responses in total. After a thorough screening process, we excluded incomplete submissions, ambiguous responses, and those completed in less than 1 minute. This rigorous filtering resulted in 327 valid questionnaires, representing an effective response rate of 78.4%.

The selection of participants leveraged snowball and convenience sampling techniques, chosen for their notable advantages, such as accessibility and the ability to reach a specific target population within a defined timeframe (Etikan et al., 2016). The demographic distribution of the sample included 27.2% male and 72.8% female students, and 66.0% of the respondents majored in business and economics (see Table 2). This composition largely reflects the recruitment channel: the instructors who assisted in distributing the survey taught blended courses concentrated in business schools. As a result, the sample should be understood as being concentrated in business and economics rather than as evenly representing all academic disciplines. The implications of this demographic imbalance for the generalizability of our findings are examined in Section 6.

**Table 2:** Demographics of respondents.

Category	Number	Percent (%)
Gender		
Male	89	27.2
Female	238	72.8
Grade		
Freshman	70	21.4
Sophomore	113	34.6
Junior	85	26.0
Senior	59	18.0
Study discipline		
Social sciences	48	14.7
Arts and humanities	9	2.8
Engineering	22	6.7
Business and economics	216	66.0
Medical sciences	4	1.2
Science	28	8.6

### 3.2 Research instrument

This study employs a meticulously designed questionnaire to collect empirical data regarding college students’ perceptions and continuance intentions toward blended learning. The questionnaire is divided into three distinct sections:

- Introduction: This initial section familiarized respondents with the concept of blended learning and common online learning platforms, such as MOOCs and the Chaoxing learning system. This introduction ensured that participants had a clear understanding of the subject matter, thereby enabling more informed responses.
- Demographic: The second section collected demographic data from respondents and established a profile of the survey participants to inform subsequent data analysis. Table 2 presents a detailed breakdown of this demographic information.
- Construct assessment: The third section was composed of questions aimed at evaluating the constructs outlined in the research model proposed for this study (see again Figure 1). The scale design encompassed eight latent variables: continuance intention (CI), perceived integration (PI), content satisfaction (CS), social satisfaction (SS), process satisfaction (PS), system functionality overload (SFO), information overload (IO), and social overload (SO).

Each construct is measured through observable variables scored on a 5-point Likert scale, ranging from 5 (Strongly Agree) to 1 (Strongly Disagree). The scales were derived from authoritative and well-established questionnaires, adapted to suit the needs of this survey. This approach ensured, to a considerable extent, the face and content validity of the instruments used. Table 1 presents a comprehensive overview of the latent factors and their corresponding survey items.

### 3.3 Data analysis and results

In the present study, Partial Least Squares Structural Equation Modeling (PLS-SEM), an evolution of Structural Equation Modeling (SEM), is employed for data analysis. PLS-SEM utilizes component-based estimation to maximize the explained variance in the dependent variable, serving to test the developed hypotheses. PLS-SEM has garnered increased scholarly attention due to its advantages: (i) It does not necessitate restrictive distributional assumptions for the data used in analysis, which is particularly beneficial given that data collected in social science research often deviates from multivariate normality; (ii) It imposes minimal constraints on sample size and residual distributions, making it an ideal choice for exploratory research and theoretical development; and (iii) When compared with the covariance-based SEM (CB-SEM) approach, PLS-SEM typically achieves a higher level of statistical power and exhibits superior convergence behavior.

Considering the exploratory nature of our research, PLS-SEM emerged as a suitable analytical method. The PLS-SEM analysis was conducted using SmartPLS 4.0 software. Adhering to the guidelines set forth by Anderson and Gerbing (1988), we employed a two-step approach to assess both the measurement and structural models.

#### 3.3.1 Measurement model

Prior to assessing the structural model, we conducted a confirmatory factor analysis (CFA) to appraise the measurement model’s sufficiency. We assessed convergent validity, a crucial aspect of measurement model validation, using three criteria outlined by Fornell and Larcker (1981): (i) item reliability, (ii) composite reliability (CR) of each construct, and (iii) average variance extracted (AVE).

For item reliability, all factor loadings needed to be statistically significant and exceed 0.50 (Hair et al., 2010). The CR, which indicates a construct’s internal consistency, was required to surpass 0.70, demonstrating reliable and consistent representation of the latent construct (DeVellis, 2003). The AVE, which measures the variance attributable to the construct beyond measurement error, was considered satisfactory at a minimum value of 0.50. This value indicates that the construct explains over half of its indicators’ variance (Fornell and Larcker, 1981).

We evaluated discriminant validity by comparing the square root of the AVE for each construct against its inter-construct correlations. Discriminant validity is established when the square root of the AVE exceeds the inter-construct correlation coefficients, indicating construct distinctiveness.

The reliability and validity of the constructs within the questionnaire were rigorously evaluated prior to model fit assessment, utilizing four indicators: Cronbach’s alpha coefficient (CA), CR, AVE, and Variance Inflation Factor (VIF). In the context of a confirmatory investigation predicated on established theories, the benchmarks for these indicators were as follows: CA should exceed 0.8, CR should be above 0.7, AVE should surpass 0.5, and VIF should approximate or be below 3 (Hair et al., 2010). The CFA results, as depicted in Table 3, were computed using SmartPLS4 software. It is gratifying to report that Cronbach’s alpha values exceeded 0.8, CR values were above 0.9, AVE scores were above 0.7, and all VIF values were below 5, indicating no serious multicollinearity concerns, although four values slightly exceeded the conservative benchmark of 3. The results, as presented in Table 3, corroborate that all eight constructs met the convergent validity criteria.

**Table 3:** Measurement model (convergent validity and reliability).

Construct	Item	Loadings	CA	CR	AVE	VIF
Perceived integration (PI)	PI1	0.927	0.912	0.945	0.851	3.460
	PI2	0.937				
	PI3	0.902				
Content satisfaction (CS)	CS1	0.919	0.929	0.949	0.823	3.247
	CS2	0.889				
	CS3	0.911				
	CS4	0.911				
Social satisfaction (SS)	SS1	0.884	0.897	0.936	0.829	3.125
	SS2	0.932				
	SS3	0.916				
Process satisfaction (PS)	PS1	0.900	0.937	0.955	0.842	3.067
	PS2	0.937				
	PS3	0.894				
	PS4	0.938				
System functionality overload (SFO)	SFO1	0.864	0.876	0.919	0.791	1.015
	SFO2	0.854				
	SFO3	0.946				
Information overload (IO)	IO1	0.859	0.931	0.912	0.722	1.014
	IO2	0.893				
	IO3	0.812				
	IO4	0.834				
Social overload (SO)	SO1	0.862	0.872	0.906	0.763	1.004
	SO2	0.903				
	SO3	0.855				
Continuance intention (CI)	CI1	0.966	0.962	0.975	0.929	DV
	CI2	0.968				
	CI3	0.959				

Further assessment of the model’s discriminant validity was conducted by comparing the square root of the AVE (denoted as SAVE) with the bivariate correlations between constructs. Fornell and Larcker (1981) posit that discriminant validity is achieved when the SAVE for each construct exceeds its inter-construct correlations. Table 4 presents these comparisons, with SAVE values surpassing the corresponding bivariate correlations, thereby affirming the discriminant validity of the model.

**Table 4:** Analysis of discriminant validity (Fornell–Larcker Criterion).

Construct	Convergent validity	Discriminant validity							
	AVE	CI	CS	IO	PI	PS	SFO	SO	SS
CI	0.929	**0.964**							
CS	0.823	0.778	**0.907**						
IO	0.722	−0.334	−0.367	**0.850**					
PI	0.851	0.81	0.817	−0.389	**0.922**				
PS	0.842	0.791	0.826	−0.383	0.798	**0.918**			
SFO	0.791	−0.16	−0.175	0.694	−0.246	−0.232	**0.889**		
SO	0.763	−0.257	−0.249	0.646	−0.369	−0.291	0.505	**0.874**	
SS	0.829	0.778	0.825	−0.364	0.78	0.912	−0.167	−0.304	**0.911**

Bold values on the diagonal represent the square roots of the average variance extracted (AVE).

#### 3.3.2 Structure model and hypotheses test

With the measurement model established as adequate, path analysis was subsequently conducted to estimate the path coefficients. The overall goodness-of-fit indices derived from the CFA suggest a model that aligns well with the data. In this study, the PLS-SEM model fit was tested by using four criteria (e.g., standard root mean square residual (SRMR), the Normed Fit Index (NFI), d_ULS and d_G). The model fit test results confirmed that the proposed model fit the data well since SRMR value (0.045) was less than 0.08, and the NFI value (0.868) was higher than the critical value of 0.8 (Henseler et al., 2016). Also, the values for d_ULS and d_G were 0.776 and 0.609, respectively, and less than 95% bootstrapped quantile (Ali et al., 2021). As such, the PLS consistent (PLSc) method was deemed the most suitable for this study.

**Figure 2:**
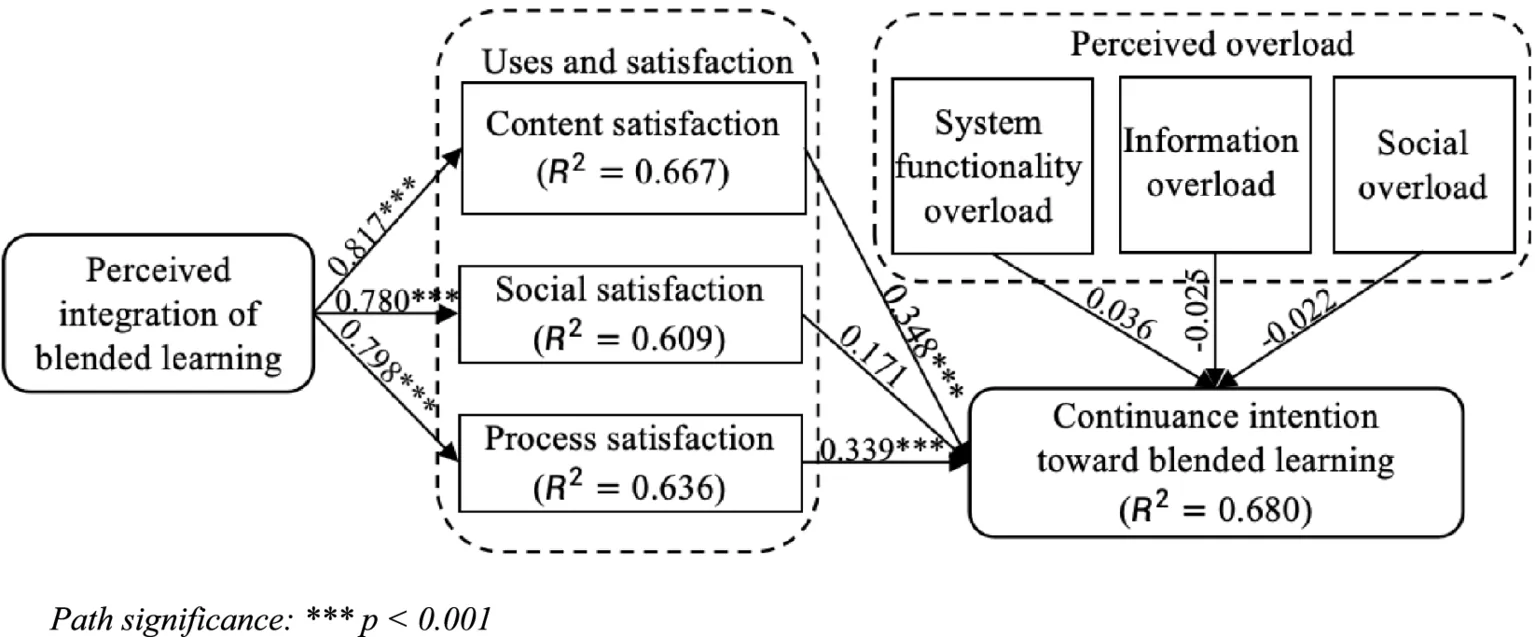
Results for the proposed research model. Path significance: ***p < 0.001.

The regression weights (β) are depicted in Figure 2, where the numerical values on the arrows represent the path coefficients between different latent constructs. According to Figure 2, the following five out of the nine formulated hypotheses were supported: PI→CS (H1), PI→SS (H3), PI→PS (H5), CS → CI (H2), and PS → CI (H6). Within the factors of uses and satisfaction affecting CI, the variables CS (*β* = 0.348, *p* < 0.001) and PS (*β* = 0.339, *p* < 0.001) demonstrated a significant effect, accounting for 68% of the variance of the CI variable; in contrast, the variable SS did not yield significant effect on CI. The results supporting hypotheses H2 and H6, in conjunction with the non-support for hypothesis H4, reveal which types of gratification influence continuance intention. Concurrently, none of the three variables within perceived overload had a significant impact on CI. The non-supported results for H7, H8, and H9 indicate that perceived overloads do not impede continuance intention in this context (see Table 5).

**Table 5:** Hypothesis testing.

Hypothesis	Path	Path coefficient	Standard deviation	*t*-values	*p*-values	Supported
H1	PI→CS	0.817	0.033	22.999	<0.001	Yes
H2	CS → CI	0.348	0.069	5.017	<0.001	Yes
H3	PI→SS	0.780	0.037	21.131	<0.001	Yes
H4	SS → CI	0.171	0.096	1.780	0.075	No
H5	PI→PS	0.798	0.035	22.744	<0.001	Yes
H6	PS → CI	0.339	0.088	3.844	<0.001	Yes
H7	SFO → CI	0.036	0.064	0.564	0.573	No
H8	IO→CI	−0.025	0.057	0.439	0.661	No
H9	SO→CI	−0.022	0.040	0.539	0.590	No

## 4 Discussion

### 4.1 Perceived integration as a gratification facilitator

Our findings provide strong empirical support for conceptualizing perceived integration as a gratification facilitator in technology-mediated learning. Perceived integration demonstrated strong effects on all three satisfaction dimensions: content satisfaction (*β* = 0.817), process satisfaction (*β* = 0.798), and social satisfaction (*β* = 0.780). These uniformly high path coefficients suggest that integration functions as a foundational environmental characteristic that enables gratification experiences across multiple psychological dimensions.

This finding extends UGT in an important way. Traditional applications of UGT focus on the gratifications users seek from media and the gratifications they obtain, with continued use depending on the match between sought and obtained gratifications (Palmgreen and Rayburn, 1982). Our results suggest that this gratification process is shaped by structural characteristics of the media environment. When learners perceive that online and offline components form a coherent, seamless whole, they are better positioned to extract content value, engage meaningfully with learning processes, and participate in social interactions. Integration, in this sense, creates the conditions for gratification rather than constituting a gratification itself.

The concept of gratification facilitator may have broader applicability beyond blended learning. In any media environment where multiple components or modalities must work together (e.g., multi-platform entertainment ecosystems, integrated health information systems, or cross-channel retail experiences), the degree of perceived integration may similarly shape users’ ability to fulfill their psychological needs. This suggests a productive direction for UGT research: examining how environmental design characteristics interact with individual gratification-seeking to determine use outcomes.

For blended learning specifically, our findings underscore that the coherence of the learning experience may be more important than the sophistication of individual components. A well-integrated system with modest features may facilitate greater gratification than a feature-rich system with poor integration. This has implications for how educational technologists conceptualize quality in blended learning design.

### 4.2 A distinct gratification pattern in educational contexts

While perceived integration enables gratification across all dimensions, these gratifications differ substantially in their influence on continuance intention. Content satisfaction and process satisfaction emerged as equally strong predictors of continuance intention (*β* = 0.348 and *β* = 0.339, respectively). This balanced influence suggests that students value both what they learn and how they learn in blended environments.

The relatively limited influence of social satisfaction (*β* = 0.171), which did not reach statistical significance at the 0.05 level, presents a theoretically provocative finding. This result directly challenges the assumption, prevalent in social media research, that social gratifications are universally important for sustained technology engagement (Gan and Wang, 2015; Ku et al., 2013). We propose that this divergence reflects fundamental differences in motivational orientation between contexts.

Rather than attributing this null effect to a single cause, we consider four complementary explanations. The first concerns motivational orientation. Drawing on Self-Determination Theory (Ryan and Deci, 2000), social media use is primarily driven by relatedness needs and intrinsic enjoyment, making social gratifications central. Educational technology use, by contrast, involves stronger achievement motivation and competence-seeking, prioritizing instrumental gratifications over social ones. This interpretation aligns with research on achievement motivation, which suggests that learners in academic contexts prioritize mastery and goal attainment over social connection (Dweck and Leggett, 1988).

The second explanation is methodological and concerns measurement. Although our social satisfaction items were adapted from established UGT scales, two of the three items, SS1 (“self-improvement”) and SS3 (“enhancing collaborative abilities”), may partly capture competence-related gratifications rather than relatedness per se. Only SS2 directly reflects community building. If the construct combines competence and relatedness elements (Ryan and Deci, 2000), its unique relationship with continuance intention may be attenuated after content and process satisfaction are taken into account. We acknowledge this measurement limitation in Section 6.

The third explanation involves functional displacement. Chinese students’ social-interaction needs are largely served by dedicated social platforms such as WeChat, whose gratification structures are well documented (Gan and Wang, 2015); meanwhile, the face-to-face component of blended learning already affords rich interpersonal contact (Garrison and Kanuka, 2004). The social features of learning platforms may therefore add little marginal social gratification, leading students to approach these platforms with a primarily task-oriented mindset.

The fourth explanation is cultural and contextual. In China’s high power-distance educational culture, formal learning settings tend to involve structured, deferential, and task-focused exchanges between teachers and students as well as among students, rather than egalitarian and open-ended (Hofstede, 1986; Zhang, 2007). Online interactions embedded in coursework, such as discussions and peer reviews, may thus be experienced as part of the academic task rather than as socially gratifying in their own right. Relatedly, because blended learning in our research context is course-embedded and its in-course use is largely arranged by instructors (see Section 3.1), much of the platform-based interaction is assigned rather than self-initiated. Such mandated interaction is unlikely to generate the voluntary sociality from which social gratification typically derives. We elaborate on this usage context in Section 4.4.

We offer these explanations as theoretically grounded but *post hoc* interpretations; they are not mutually exclusive, and future research should adjudicate among them, for example by refining the measurement of social gratification or by comparing voluntary and mandatory usage settings (see Section 6).

These findings suggest that gratification structures are context-dependent: the relative salience of gratifications that drives continued use varies systematically across media domains. For UGT, this implies that researchers should not assume that gratification patterns observed in one context (e.g., social media) will generalize to others (e.g., educational technology). Context-specific gratification patterns may need to be empirically established rather than theoretically assumed.

### 4.3 Resilience to cognitive overload

Diverging from the detrimental effects often associated with CLT (Sweller, 1988; Kirschner, 2002), system functionality overload, information overload, and social overload did not significantly affect continuance intention. This null finding is theoretically important because it suggests boundary conditions for cognitive load effects.

We propose two complementary explanations. First, from a developmental perspective, contemporary university students have extensive experience navigating complex information environments. This prolonged exposure may have fostered enhanced cognitive schemas for managing digital complexity, effectively raising the threshold at which overload impairs engagement.

Second, from a motivational perspective, Expectancy-Value Theory (Wigfield and Eccles, 2000) suggests that individuals persist with challenging tasks when perceived value outweighs perceived costs. Our participants may have developed compensatory strategies such as selective attention, strategic information filtering, distributed processing across time to mitigate overload effects when motivation for learning outcomes is sufficiently high.

It is also worth noting that our measure of perceived integration may have absorbed some variance that might otherwise manifest as overload effects. When students perceive high integration, they may experience the learning environment as more manageable and coherent, reducing the subjective experience of overload. Future research could examine whether integration moderates the relationship between objective complexity and perceived overload.

These findings contribute to an emerging literature questioning whether cognitive load effects generalize uniformly across populations and contexts (de Jong, 2010). Future research should examine whether overload tolerance varies with digital experience, motivation levels, or individual differences in working memory capacity.

### 4.4 Contextual considerations

Our study was conducted among Chinese university students in the post-pandemic educational landscape. The COVID-19 pandemic accelerated the adoption of blended learning and may have shaped students’ expectations and adaptive capabilities. Students who have navigated emergency remote learning may have developed greater tolerance for technology-mediated education and more sophisticated strategies for managing its demands.

The specific cultural and institutional context may also influence our findings. Chinese higher education emphasizes academic achievement and self-directed learning, which may amplify the importance of content and process satisfaction. Additionally, China’s advanced digital infrastructure and widespread technology adoption have produced a student population highly adapted to complex digital environments.

A further contextual feature is the quasi-mandatory nature of usage. As described in Section 3.1, the in-course use of blended learning components is arranged by instructors, whereas our dependent variable captures students’ volitional intention to continue with blended learning beyond such requirements. This context carries two interpretive implications. First, gratifications obtained under partly mandated use may differ from those arising in fully voluntary settings: assigned interactions, in particular, may mute the social gratification that voluntary interaction would otherwise generate, consistent with the non-significant effect of social satisfaction (Section 4.2). Second, our findings speak to volitional continuance with blended learning as a mode of study; they should be generalized with caution to settings where continued platform use is itself institutionally compelled, since behavioral intention plays a weaker role in mandated environments (Venkatesh et al., 2003).

These contextual factors delineate which of our findings are likely to travel and which require situated replication. One potentially transferable finding is the strong relationship between perceived integration and the three satisfaction dimensions. This relationship may extend beyond China and higher education: any learning environment that combines online and offline components faces the same coherence problem, and the gratification-facilitator logic may apply wherever multiple modalities must work together (see also Section 4.1). By contrast, the relative weights of the gratifications are plausibly context-bound: in cultures with lower power distance, in discussion-based disciplines, or in fully voluntary usage settings, social satisfaction may well regain the predictive power it lacks here, just as overload effects may emerge among less digitally experienced populations. Cross-cultural and cross-level replication is therefore not merely desirable but the natural next test of the framework.

## 5 Implications

### 5.1 Theoretical implications

*Introducing the gratification facilitator concept*: Our findings demonstrate that perceived integration functions as a powerful antecedent of gratification, strongly predicting content, process, and social satisfaction (*β* = 0.78–0.82). This introduces the concept of gratification facilitator (an environmental design characteristic that shapes users’ ability to fulfill psychological needs) to UGT. This extension suggests that UGT should incorporate not only gratifications sought and obtained, but also the structural properties of media environments that enable gratification experiences.

*Revealing context-dependent gratification structures*: The distinct gratification structure observed in educational technology, where cognitive and instrumental gratifications dominate over social gratifications, challenges assumptions derived from social media research. This finding suggests that motivational orientation (achievement vs. social) may moderate which gratifications become salient, calling for context-specific applications of UGT across different technology domains.

*Identifying boundary conditions for cognitive load effects*: The absence of overload effects on continuance intention suggests that cognitive load predictions may be bounded by individual differences in digital experience and motivational intensity. This points toward integrated models that consider both cognitive capacity constraints and motivational factors in predicting technology engagement.

### 5.2 Practical implications

The strong influence of perceived integration on student satisfaction highlights the need for educators and institutions to focus on creating seamless blended learning experiences. This involves ensuring content integration between online and offline materials, implementing learning management systems that effortlessly connect various components, developing teaching strategies that effectively combine online and face-to-face methodologies, designing integrated assessment methods, and providing comprehensive support systems. By enhancing integration across these dimensions, institutions can create a more cohesive and satisfying learning experience for students.

Given the significant impact of content and process satisfaction on continuance intention, educators and instructional designers should prioritize the development of high-quality, diverse, and engaging learning materials. Equally important is the design of intuitive and effective learning processes that guide students through the course content. Regular updates and refinements based on student feedback and learning analytics can ensure that the content remains relevant and the learning process remains effective. Implementing interactive elements such as simulations, adaptive learning paths, and immediate feedback mechanisms can further enhance the learning process and boost student satisfaction.

While our findings suggest that social features may not be as critical for driving continued use as previously thought, they should not be entirely neglected. Instead of investing extensive resources in developing complex social interaction tools within learning platforms, educators should focus on creating meaningful, task-oriented collaborative activities that enhance learning outcomes. Utilizing existing social platforms that students are familiar with for course-related social interactions and ensuring that face-to-face components provide ample opportunities for community building can be more effective strategies.

The resilience of students to perceived overloads suggests that educators can design more sophisticated and feature-rich learning environments without fear of overwhelming students. This opens up possibilities for incorporating a wider range of digital tools and resources to enhance the learning experience. Educators can encourage students to explore advanced features of learning platforms, promoting digital literacy and self-directed learning. However, it is crucial to maintain a balance and ensure that added complexity serves a clear pedagogical purpose.

For educational technology developers, our findings reframe what constitutes product quality. The dominant driver of student satisfaction in our data was not feature richness but perceived integration—how coherently a platform’s online components mesh with offline instruction. Developers should therefore prioritize interoperability and workflow continuity over feature accumulation: seamless handoffs between classroom content and platform resources (e.g., automatically linking lecture materials to follow-up online tasks), synchronization between in-class activities and online assignments, and compatibility with the tools instructors already use. By contrast, heavy investment in elaborate in-platform social networking features appears less likely to pay off in educational settings; lightweight, task-oriented collaboration tools may suffice.

For policymakers, university administrators, and industry stakeholders, the results caution against evaluating blended learning initiatives by counting digital features or platform adoption rates. Quality assurance frameworks, funding criteria, and procurement standards should instead assess integration quality, which refers to the coherence students experience between online and offline components. Because such coherence is created pedagogically as much as technically, policies that fund instructor training in blended course design may yield greater returns than policies that fund platform features alone. Beyond higher education, the gratification-facilitator logic extends to corporate training and lifelong learning programs that combine digital and face-to-face elements: providers in these settings should likewise prioritize the design and evaluation of cross-modality coherence rather than the sophistication of individual components.

When implementing blended learning in different cultural contexts, it is important to consider local educational norms and expectations. The balance between online and offline components may need to be adjusted to suit cultural preferences and technological infrastructure. Regular surveys and focus groups can help institutions understand and respond to culture-specific student needs and preferences, ensuring that the blended learning approach is both effective and culturally appropriate.

Finally, institutions should leverage the power of learning analytics to continuously improve their blended learning offerings. Implementing robust analytics systems can provide valuable insights into student engagement, satisfaction, and performance. These data-driven insights can inform the ongoing refinement of online and offline components, leading to more personalized and effective learning experiences. By adopting a culture of continuous improvement based on analytics, institutions can ensure that their blended learning offerings remain relevant, engaging, and effective in meeting student needs.

## 6 Limitations and directions for future research

While our study provides valuable insights into the factors associated with students’ continuance intention in blended learning, several limitations should be acknowledged:

1. The study’s geographical scope was limited to Chengdu, China, which limits the generalizability of the findings. Future research should conduct comparative studies across different regions and countries to examine cultural influences on blended learning perceptions and use.
2. Relatedly, the sample is demographically imbalanced: female students (72.8%) and business and economics majors (66.0%) are overrepresented, reflecting the convenience and snowball sampling procedure described in Section 3.1. Therefore, the findings should be generalized with caution beyond business and economics students in Chinese higher education. Such imbalance does not invalidate the structural relationships estimated within the sample, but it may constrain the generalizability of our findings, particularly if gender or disciplinary background moderates the hypothesized paths, as prior research on technology acceptance suggests is possible (Padilla-Meléndez et al., 2013; Khechine et al., 2014). Future research should replicate the model with quota or stratified samples that balance gender and academic disciplines.
3. Our reliance on self-reported data may introduce bias. Future studies should incorporate objective measures of student engagement and performance alongside self-reported data to provide a more comprehensive understanding of blended learning effectiveness.
4. The non-significant relationship between social satisfaction and continuance intention warrants further investigation. As discussed in Section 4.2, SS1 and SS3 may partly capture competence-related rather than relational gratifications, meaning that the construct may combine two distinct need dimensions. Future research should develop measures that distinguish relational gratifications, such as belongingness and community, from competence-oriented gratifications. Future studies should also examine how the quality and quantity of social activities influence student engagement across voluntary and mandatory interaction settings.
5. Our treatment of perceived integration also warrants refinement. The three measurement items address different aspects of integration. PI1 captures content integration, PI2 captures activity complementarity, and PI3 captures spatiotemporal extension. In the present study, these items were modeled as indicators of a single first-order construct. Because each aspect is represented by only one item, the current data do not support reliable estimation of a multidimensional or higher-order model. The retained specification nevertheless demonstrates strong reliability and convergent validity (loadings = 0.902–0.937; CR = 0.945; AVE = 0.851). However, aggregating the three aspects may obscure their differential relationships with content, process, and social gratifications. Future research should develop multiple indicators for each aspect, compare unidimensional, correlated multidimensional, and higher-order specifications, and test the effects of each aspect on the three gratification dimensions.
6. Although our model links perceived integration to three satisfaction dimensions and continuance intention, it does not estimate indirect effects or include additional cognitive, affective, and motivational variables. The findings therefore describe the hypothesized structural relationships rather than the full internal process through which perceived integration relates to continuance intention. Future research should estimate these indirect effects and examine variables such as perceived usefulness, enjoyment, engagement, and self-efficacy. Longitudinal or experimental designs would also help establish temporal order.
7. While our study found that perceived overloads did not significantly impact continuance intention, future research could investigate the adaptive strategies students may have developed to manage potential overloads in digital learning environments.
8. Finally, given the rapid pace of technological change, future studies should examine how emerging technologies such as generative AI tools, AI-based adaptive learning systems, and immersive virtual reality reshape the blended learning experience. Recent research on generative AI in education, including ChatGPT-assisted blended learning, illustrates how new technologies add components to already multi-component learning environments (Lee et al., 2024; Liang et al., 2026). This makes the coherence question at the heart of this study more pressing rather than less: whether perceived integration retains, or even strengthens, its gratification-facilitating role in AI-enhanced blended learning is a natural extension of our framework.

## 7 Conclusion

This study extends UGT to educational technology contexts, with two primary theoretical contributions. First, we introduce and validate perceived integration as a gratification facilitator, an environmental design characteristic that strongly predicts gratification across content, process, and social dimensions (*β* = 0.78–0.82). This finding suggests that UGT should be extended to incorporate not only the gratifications users seek and obtain, but also the structural properties of media environments that enable gratification experiences. In blended learning, the coherence between online and offline components creates the conditions for need fulfillment, underscoring that how well system components work together may matter more than the sophistication of individual features.

Second, we reveal a distinct gratification pattern in educational technology that differs from patterns observed in social media research. Reflecting cognitive and instrumental gratifications, content satisfaction and process satisfaction significantly predict continuance intention, while social satisfaction shows no significant effect. This pattern suggests that gratification structures are context-dependent, with educational settings prioritizing achievement-oriented over social-affective gratifications. The finding challenges assumptions about the universal importance of social features in technology engagement and calls for context-specific applications of UGT.

Additionally, our finding that perceived overloads do not impede continuance intention highlights potential boundary conditions for CLT to contemporary learners. This resilience likely reflects extensive experience with digital environments and the development of adaptive coping strategies, suggesting boundary conditions for cognitive load effects that warrant further investigation.

For educational practice, these findings suggest that institutions should prioritize integration quality over feature accumulation, focus resources on content and process design, and leverage students’ digital proficiency to create rich learning environments. The seamless coherence of the blended learning experience emerges as the key design priority for sustaining student engagement, not the complexity of social features or concerns about information overload.

As technology-mediated learning continues to evolve, understanding the factors associated with students’ continuance intention becomes increasingly important. Our study contributes to this understanding by demonstrating that what students seek from educational technology is, fundamentally, a well-integrated environment that supports effective pursuit of their learning goals.

## Data Availability

The raw data supporting the conclusions of this article will be made available by the authors, without undue reservation.
